# Entomopathogenic Fungi in Biological Plant Protection: The Machinery of Multicomponent System Interactions

**DOI:** 10.3390/jof9080825

**Published:** 2023-08-04

**Authors:** Ivan M. Dubovskiy, Tariq Butt

**Affiliations:** 1 Laboratory of Biological Plant Protection and Biotechnology, Novosibirsk State Agrarian University, Dobrolubova Str. 160, 630039 Novosibirsk, Russia; 2 Laboratory of Biotechnology of Microorganisms and Plants, Tomsk State University, 634050 Tomsk, Russia; 3 Siberian Federal Scientific Centre of Agro-BioTechnologies of the Russian Academy of Sciences, 630501 Krasnoobsk, Russia; 4 Department of Biosciences, Swansea University, Singleton Park, Swansea SA2 PP, UK; t.butt@swansea.ac.uk

Plant protection faces a growing number of challenges, partly stemming from intensification of plant cultivation to ensure food security for a rapidly growing global population. The challenges include extensive and widespread use of pesticides and fertilizers which pose a risk to human health and damage the environment. The challenges are exacerbated by the development of resistance in pest populations, withdrawal of many conventional pesticides, and the threat of invasive species. One solution to these challenges is the use of entomopathogenic fungi (EPF), particularly members of the Hypocreales such as *Metarhizium*, *Beauveria* and *Isaria*. Efficacious use of these microbes in plant protection programmes requires an in-depth understanding of their ecology, evolution and biology, especially interactions with the invertebrate host [1,2]. Currently over 700 EPF-based products are sold worldwide. The market share is increasing and will continue to increase as more growers become familiar with these organisms, but also because these fungi have been shown to stimulate plant growth and increase their resistance to biotic and abiotic stress.

This Special Issue attracted high-quality articles addressing both fundamental and applied questions pertinent to the development EPF for use in crop pest management programmes. These studies, conducted at the molecular-biochemical to whole organism level, revealed a myriad of diverse, complex interactions which have greatly enhanced our understanding of EPF and how best to exploit them or their byproducts. Researchers established that the Slt2-MAPK signalling pathway and the transcription factor RNS1 controlled conidiation in *Metarhizium robertsii* via direct regulation of the central regulatory pathway [3]. They also identified the elongator subunit (Elp3) that regulates development, stress tolerance, cell cycle and virulence in *Beauveria bassiana* [4]. MicroRNAs involved in immunity and development were found to participate in the modulation of *M. anisopliae–Plutella xylostella* interactions [5]. Toll-like receptors (TLRs) in *Diaphorina citri* were shown to be induced by endophytic *B. bassiana* [6]. *Lobesia botrana* pupae were shown to be susceptible to *Beauveria pseudobassiana* with those without a cocoon being more susceptible than those with a cocoon [7].

Studies of EPF–insects and EPF–plant systems in field conditions have helped improve deployment of EPF. For example, direct spraying of *B. bassiana* significantly reduced survival of all life stages of the green stink bug, *Nezara viridula* [8]. Researchers have shown that resporulated *M. anisopliae* granules were highly infective, causing 100% mortality of *Tenebrio molitor* larvae 9 days post inoculation [9]. Much progress has been made in the production of *Metarhizium* microsclerotia, and identification of factors affecting their storage and germination [10]. EPF coformulation with nanoparticles (bypassing host defence systems) and dsRNA (inducing RNAi) were shown to enhance EPF efficacy in pest control [11,12]. Combinations of *B. bassiana* and emamectin benzoate were shown to act synergistically, providing significantly higher control of *Megalurothrips usitatus* than if either agent was used alone [13]. Root colonization by *M. brunneum* was shown to prime local and systemic jasmonic acid pathways in oilseed rape *Brassica napus*, thus negatively affecting the development of cabbage root fly (*Delia radicum)* larvae [14].

There were several studies showing that EPF stimulating plant grow, and some studies shed light on the underlying mechanisms. Six *M. anisopliae* CFEM proteins with various structures were analysed in connection to EPF-plant interactions and their subcellular localization in host cells suggests they play some role during plant colonization by the fungus [15]. Furthermore, *B. bassiana-* and *M. brunneum*-mediated Fe solubilization was shown to involve upregulation of Fe acquisition genes in melon and cucumber [16]. Researchers also showed that onion plants colonized by endophytic *B. bassiana* were more tolerant to stress due to drought [17]. New studies highlight that *M. brunneum* volatile organic compounds (VOCs) were actively involved in plant growth promotion of a wide range of commercially important crops [18]. These VOCs were also shown to suppress the development of bacterial and plant pathogenic fungi [19].

We would like to thank all contributors to this Special Issue on “Entomopathogenic fungi in biological plant protection: The machinery of multicomponent system interactions” for their significant contributions to this Special Issue and for making it a highly successful and timely collection of studies.

## Data Availability

Data sharing not applicable.
